# Immune Complex Glomerulonephritis in a Patient with Myelodysplastic Syndrome with Ring Sideroblasts Treated with Luspatercept

**DOI:** 10.3390/diagnostics13010011

**Published:** 2022-12-21

**Authors:** Sigurd Delanghe, Tri Q. Nguyen, Dominiek Mazure, Amélie Dendooven, Marijn M. Speeckaert

**Affiliations:** 1 Department of Nephrology, Ghent University Hospital, 9000 Ghent, Belgium; 2 Department of Pathology, University Medical Center Utrecht, 3584 CX Utrecht, The Netherlands; 3 Department of Hematology, Ghent University Hospital, 9000 Ghent, Belgium; 4 Department of Pathology, Ghent University Hospital, 9000 Ghent, Belgium; 5 Faculty of Medicine and Health Sciences, University of Antwerp, 2000 Antwerp, Belgium; 6 Research Foundation-Flanders (FWO), 1090 Brussels, Belgium

**Keywords:** myelodysplastic syndrome with ring sideroblasts, acute kidney injury, immune-complex-mediated proliferative glomerulonephritis, transforming growth factor-beta, Smads

## Abstract

Myelodysplastic syndromes (MDS) are a group of clonal myeloid disorders distinguished by dysplastic bone marrow and peripheral blood cells, ineffective hematopoiesis, and an increased risk of developing acute myeloid leukemia (AML). MDS with ring sideroblasts (MDS-RS) is a favorable outcome subtype with a lower frequency of AML transformation. The FDA recently approved luspatercept for the treatment of patients with very-low-, low-, and intermediate-risk MDS-RS who have failed to correct anemia with an erythropoiesis-stimulating agent (ESA) and require two units of red blood cells over an eight-week period. This drug’s pharmacology is based on the critical role of the transforming growth factor-beta (TGF-β) pathway in regulating erythropoiesis. In this case report, we describe for the first time an acute kidney injury caused by membranoproliferative glomerulonephritis (MPGN) in a patient with MDS-RS who was treated with luspatercept. We propose that a multi-hit hypothesis could explain the immunopathogenesis. A first unknown hit may stimulate IgA immune complex production, whereas luspatercept administration acts as a second hit, causing Smad1-5-8 phosphorylation. This intriguing case report on immune-complex-mediated proliferative glomerulonephritis following luspatercept treatment generates hypotheses and stimulates further research in this area.

## 1. Introduction

Myelodysplastic syndromes (MDS) are a diverse group of clonal myeloid disorders, which are distinguished by a dysplastic bone marrow and peripheral blood cells, an ineffective hematopoiesis, and an increased risk of developing acute myeloid leukemia (AML). MDS with ring sideroblasts (MDS-RS) is a subtype with a favorable outcome and a lower frequency of AML transformation [1]. Anemia is the first symptom in approximately 80% of lower-risk MDS patients. Current treatment options for patients with lower-risk MDS are aimed at correcting cytopenias, reducing anemia and transfusion burden, and preventing complications such as infections or bleeding [2].

MDS is associated with a high prevalence of autoimmune manifestations reported in 10–20% of patients [3]. Although kidney involvement is rarely reported, a broad spectrum of glomerular diseases has been described in association with MDS [4]. A recent case series described tubulointerstitial nephritis as the most prevalent lesion [3] although mesangial proliferative glomerulonephritis, immunoglobulin-associated membranoproliferative glomerulonephritis (MPGN), IgA nephropathy, IgA vasculitis, C3 glomerulopathy, fibrillary glomerulonephritis, focal segmental glomerulosclerosis, minimal change disease, and membranous nephropathy have also been described in patients with MDS [4]. 

Recently, luspatercept has been approved by the FDA for treatment of patients with very-low-, low-, and intermediate-risk MDS-RS (or MDS/myeloproliferative neoplasm (MPN) with RS and thrombocytosis) who have failed to correct anemia with an erythropoiesis-stimulating agent (ESA) and require ≥ 2 units of red blood cells over eight weeks [5]. The pharmacology of this drug is based on the important role of the transforming growth factor beta (TGF-β) pathway in regulating erythropoiesis. Luspatercept is an activin receptor type IIB (ActRII) fusion ligand trap agent. The trap consists of an extracellular receptor domain fused to the Fc portion of an IgG1 domain. It neutralizes the ligands before binding the receptor and thus inhibits signaling through the receptor [6]. Luspatercept binds to growth differentiation factor (GDF) 8 and 11, activin B, and other ligands, but the exact contribution of each ligand to the pharmacodynamic effect of luspatercept has not yet been determined. This binding leads to inhibition of Smad 2/3 signaling, which is known to be abnormally high in disease models of ineffective erythropoiesis such as MDS and beta-thalassemia, resulting in erythroid maturation and differentiation [7]. Lusparcept binding with its ligand leads also to an upregulation of GATA1 (GATA Binding Protein 1) and its target gene signature in erythroid precursors [8]. In contrast to erythropoietin, which acts mainly on colony-forming units-erythroid (CFU-Es), ActRII ligand traps stimulate late erythroid precursors [9]. Apart from the erythroid precursors, the bone marrow microenvironment in MDS patients could be potentially altered by affecting collagen production and other components in the extracellular membrane by mesenchymal stromal cells. 

Because the TGF-β pathway affects multiple organ systems throughout the body, there is a risk that luspatercept will have off-target effects [10]. Kidney injury was observed in 10% of MDS patients who received luspatercept compared to 6.6% in the placebo group [7]. Luspatercept has been shown to cause a membranoproliferative pattern of glomerular injury in rats and monkeys in non-clinical studies (FDA and EMA pre-clinical data) [11,12], but this has never been demonstrated in human subjects. In this case report, we describe for the first time an acute kidney injury caused by MPGN in an MDS-RS patient treated with luspatercept.

## 2. Case Description

**Figure 1:**
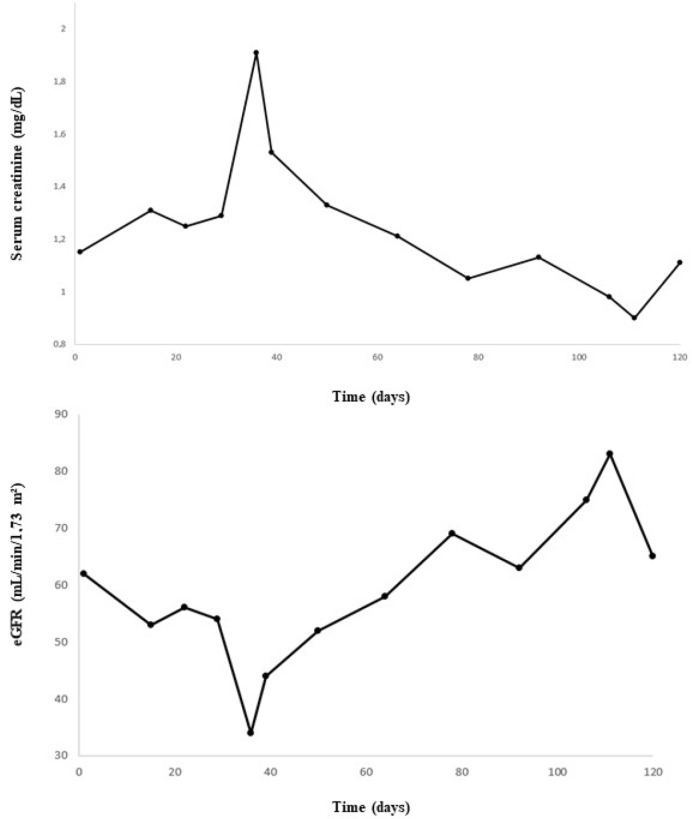
Evolution of serum creatinine and estimated glomerular filtration rate (eGFR) over time.

**Figure 2:**
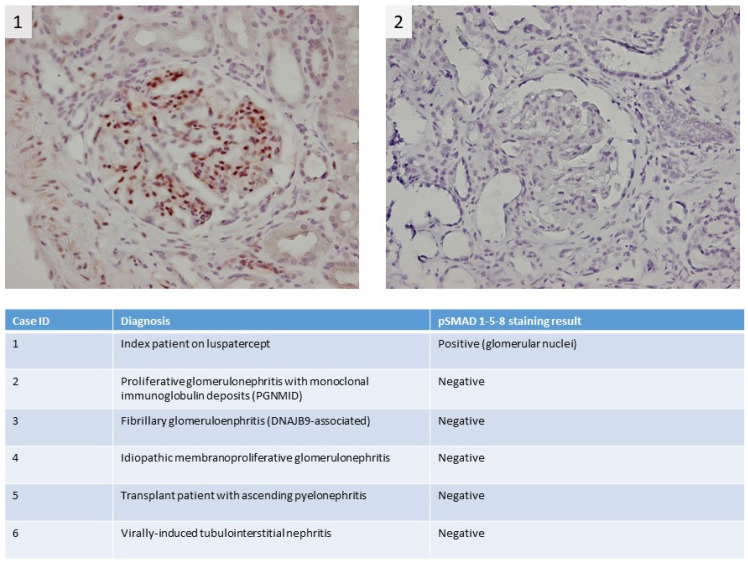
Immunohistochemical pSMAD 1-5-8 staining shows strong staining in the kidney of the myelodysplastic syndrome with ring sideroblasts (MDS-RS) patient; more specifically, there is clear positivity in the nuclei of the glomerular cells, which indicates activation of the pSMAD 1-5-8 pathway (**1**). For comparison, the staining results of a control patient with a membranoproliferative pattern due to proliferative glomerulonephritis with monoclonal immune deposits (PGNMID) are depicted in the right panel (**2**). In the table, staining results for pSMAD 1-5-8 in additional control patients are summarized.

A 74-year-old male patient with MDS-RS and with multilineage dysplasia (MDS-RS-MLD with mutations in ASXL1 and SF3B1, IPSS score = 0 (low risk), IPSS-R score = 3.5 (intermediate risk)) was referred to the Department of Nephrology due to an acute kidney injury. He was also treated for arterial hypertension and chronic obstructive pulmonary disease at the time (COPD). In the past, he underwent a hemicolectomy because of multiple adenomatous polyps. In 2018, a prostatectomy with lymphadenectomy was performed as he suffered from prostate cancer (Gleason score = 7), which was followed by adjuvant radiotherapy. Recently, because of transfusion-dependent anemia (hemoglobin: 7.9 g/dL) with an erythropoietin level of 1735 IU/L (4.3–29.0 IU/L) due to MDS-RS-MLD, luspatercept was started at a dose of 1 mg/kg every 3 weeks. A progressive decline in kidney function was observed (Figure 1), starting with a baseline serum creatinine of 0.99 mg/dL (eGFR CKD-EPI: 74 mL/min/1.73 m^2^) and with a maximum increase in serum creatinine to 1.91 mg/dL (eGFR CKD-EPI: 34 mL/min/1.73 m^2^) 1.5 months after the first administration of luspatercept. Urinalysis revealed leukocyturia, microscopic hematuria, pathologic casts, and proteinuria in the nephrotic range of 4.63 g/g creatinine. No previous urinalysis in the months before starting luspatercept was available. Anti-nuclear factor (ANF), antineutrophil cytoplasmic antibodies (ANCA), and anti-glomerular basement membrane (anti-GBM) antibodies were all negative in immunological testing. Serum albumin and complement levels were normal. In a polyclonal background, a serum protein electrophoresis revealed a very weak banding in IgG kappa. A kidney ultrasound was performed, which showed a normal size of the kidneys: the length of the right kidney was 12.0 cm and that of the left kidney 11.4 cm. There was mildly impaired corticomedullary differentiation at both sides. No hydronephrosis was observed. After stopping the administration of luspatercept, a spontaneous recuperation of the kidney function was observed. However, as the urinary abnormalities persisted (pyuria, microscopic hematuria, pathologic casts (grain cylinders), and proteinuria of 3.22 g/g creatinine), a kidney biopsy was performed two months after the start of luspatercept. This showed an image of an IgA-dominant immune complex-mediated glomerulonephritis (with membranoproliferative pattern and clear endocapillary proliferation). Because there was a strong suspicion that the membranoproliferative glomerulonephritis was caused by luspatercept, ancillary immunohistochemistry for pSmad1-5-8 (Cell Signaling Technology; Danvers, MA, USA) was performed. This showed positive results in our patient’s glomeruli, but the immunohistochemistry for pSmad1-5-8 was negative in the six renal biopsies of patients with various other glomerulopathies (Figure 2). Immunohistochemistry for Smad2/3 (Cell Signaling) was unable to distinguish between renal pathology in our patients and in our control population (data not shown). Methylprednisolone 64 mg daily was started and tapered over the next 12 weeks, accompanied by a gradual improvement in urinary sediment and slow resolution of proteinuria.

## 3. Discussion

We present a case of membranoproliferative glomerulonephritis (MPGN), probably secondary to luspatercept in a patient with MDS-RS. The reasons for the link between this drug and the MPGN pattern are the temporal relationship between the start of the drug and the rise in serum creatinine, the positive staining for pSmad1-5-8 on renal biopsy (which was absent in the control population), and finally the EMA/FDA preclinical data about the presence of MPGN in rhesus monkeys and rats. According to the EMA11 and FDA data [13], ACE-536 was administered at dosages of 0.3, 1, and 6 mg/kg (5/sex/group) in a 6-month toxicity study (20039148) with Cynomolgus monkeys. The effects on renal changes included a dose-dependent minimal increase in creatinine in males and females at 1 mg/kg, a dose-dependent minimal increase in BUN at 6 mg/kg, and histopathology changes in the kidney consisting of MPGN in males and females at 1 mg/kg. Other effects included tubular protein accumulation, interstitial or tubular hemorrhage, interstitial fibrosis/fibroplasia, increased extracellular matrix, vacuolization of interstitial cells, and/or degeneration/atrophy of tubules in the medulla near the corticomedullary junction as well as interstitial mixed inflammatory cell infiltrates.

**Figure 3:**
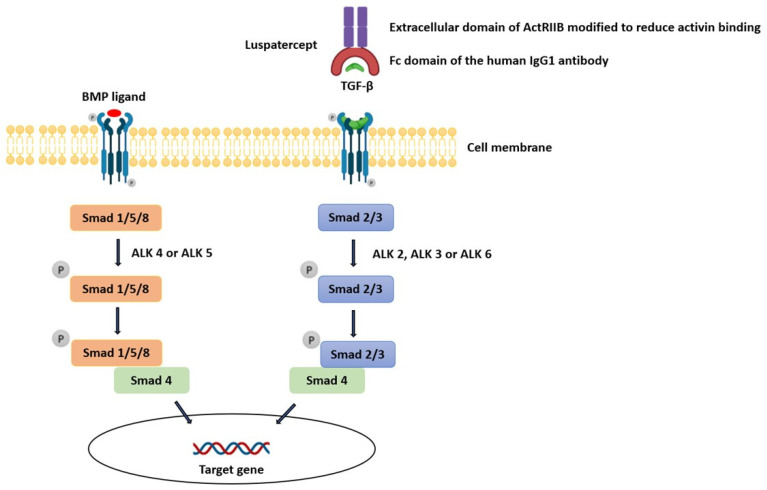
Influence of luspatercept on the TGF-β pathway.

The linkage between TGF-β inhibition, which is the mechanism by which luspatercept exerts its action, and the development of an auto-immune disease (e.g., immune-complex-mediated proliferative glomerulonephritis) is complex. TGF-β proteins are part of a large family of secreted growth factors with pleiotropic activities in diverse tissues ranging from proliferation, cell death, and differentiation to lineage determination, organ morphogenesis, and tissue homeostasis [14]. The TGF-β family comprises over 30 members: subclasses include TGF-Betas (TGF-β1, TGF-β2, and TGF-β3), bone morphogenic proteins (BMPs), activins, and growth differentiation factors (GDFs). Ligands can bind to twelve different TGF-β family receptors, which include seven type I receptors (ALK1-7) and five type II receptors (TGF-βRII, BMPRII, ActRII, ActRIIB, and AMHRII), and different TGF-β ligands bind different combinations of type I and type II receptors. A type I and II receptor typically dimerize together along with its specific ligand, forming a complex. This produces signals of remarkable complexity which then activate one of the Smad pathways. Smads are a family of cytoplasmic proteins that are responsible for downstream effector signaling in the TGF-β pathway. TGF-β, Nodal, and Activin generally induce phosphorylation of Smad2 and Smad3 via ALK4 or ALK5, whereas BMPs phosphorylate Smad1, Smad5, or Smad8 through ALK2, ALK3, or ALK6 (Figure 3). In endothelial cells, however, TGF-β can bind receptor complexes containing either ALK5 (TβRI) or ALK1 (ACVRL1), activating Smad2 and Smad3 via ALK5 but also Smad1 and Smad5 via ALK1. In addition to the canonical Smad-mediated pathways, there are several non-Smad mediators of TGF-β signaling, including c-Jun amino-terminal kinase (JNK), p38 mitogen-activated protein kinase (MAPK), Akt, and others, collectively known as non-canonical signaling mediators [15].

In general, the TGF-β pathway is thought to be myelosuppressive in nature [7]. TGF-β regulates the generation and effector functions of many immune cell types in both the adaptive and innate immune system. It controls adaptive immunity by directly promoting the expansion of regulatory T cells (Treg cells) and by inhibiting the generation and function of effector T cells and antigen-presenting dendritic cells. TGF-β mediates IgA class switching and mediates transition from acute inflammation to fibrotic tissue healing and repair via Treg cells. TGF-β similarly controls the innate immune system by inhibiting natural killer cells and regulating the complex behavior of macrophages and neutrophils and thus causing negative immune regulatory inputs [16]. Additionally, activin A is known to play a role in immunoregulation and signals through Smad2/3 [17].

One of the most well-characterized roles of TGF-β is its induction of fibrosis in most organs, and in this way, it is a major contributor to progressive CKD regardless of the etiology [18,19]. Smad3 inhibition and global ablation in mice afford protection from fibrosis in response to unilateral ureteral obstruction, diabetes mellitus, and renal toxins. Conditional renal tubular-specific up-regulation of TGF-β1, moreover, is sufficient to induce renal fibrosis, confirming the pathogenic role of the TGF-β1/Smad3 pathway in CKD [20].

The Smad pathway that appears most important for hematopoiesis includes signaling through the Smad2/3 heterodimer (whose activation is repressed by Smad7), leading to activation and nuclear localization of Smad4 or transcription intermediary factor 1-gamma (TIF1-γ), which can then trigger competing transcription paradigms to permit growth inhibition or erythroid differentiation, respectively [8,10,15]. In this subfamily, Smad1, Smad5, and Smad8 act as positive signal transducers activated by interaction with ligand-activated BMP receptors, whereas Smad2 and Smad3 act in the TGF-β signaling. BMP signaling via Smad1/5/8 complex can counter-regulate TGF-β/Smad-mediated renal fibrosis [8,14].

Because the precise contribution of each ligand to the effect of luspatercept is unknown, the underlying pathophysiology can only be hypothesized. Combining our current knowledge about the TGF-β pathway, the preclinical data, and the pathology report, it can be hypothesized that luspatercept causes an overactivation of the Smad1-5-8 pathway, which could lead to the clinical picture seen in our patient. The IgA dominance on immunofluorescence cannot be readily explained, but it is known that Smads play a role in IgA class switching. As mentioned before, TGF-β and its family form a complex network of receptors and are implicated in a wide range of immunological processes. 

## 4. Conclusions

In the present case, we propose that the immunopathogenesis could be explained by a multi-hit hypothesis. A first unknown hit may stimulate the production of IgA immune complexes, whereas luspatercept administration acts as a second hit, causing Smad1-5-8 phosphorylation. In conclusion, this interesting case report on immune-complex-mediated proliferative glomerulonephritis after treatment with luspatercept is hypothesis-generating and stimulates further research in this area.

## Data Availability

Not applicable.
